# COMPare: Qualitative analysis of researchers’ responses to critical correspondence on a cohort of 58 misreported trials

**DOI:** 10.1186/s13063-019-3172-3

**Published:** 2019-02-14

**Authors:** Ben Goldacre, Henry Drysdale, Cicely Marston, Kamal R. Mahtani, Aaron Dale, Ioan Milosevic, Eirion Slade, Philip Hartley, Carl Heneghan

**Affiliations:** 1 Centre for Evidence-Based Medicine, Department of Primary Care Health Sciences, University of Oxford, Radcliffe Observatory Quarter, Woodstock Road, Oxford, OX2 6GG UK; 2 Department of Social and Environmental Health Research, London School of Hygiene and Tropical Medicine, Keppel Street, London, WC1E 7HT UK

**Keywords:** Outcomes, Misreporting, Trials, CONSORT, Audit, Correction letters

## Abstract

**Background:**

Discrepancies between pre-specified and reported outcomes are an important and prevalent source of bias in clinical trials. COMPare (Centre for Evidence-Based Medicine Outcome Monitoring Project) monitored all trials in five leading journals for correct outcome reporting, submitted correction letters on all misreported trials in real time, and then monitored responses from editors and trialists. From the trialists’ responses, we aimed to answer two related questions. First, what can trialists’ responses to corrections on their own misreported trials tell us about trialists’ knowledge of correct outcome reporting? Second, what can a cohort of responses to a standardised correction letter tell us about how researchers respond to systematic critical post-publication peer review?

**Methods:**

All correspondence from trialists, published by journals in response to a correction letter from COMPare, was filed and indexed. We analysed the letters qualitatively and identified key themes in researchers’ errors about correct outcome reporting, and approaches taken by researchers when their work was criticised.

**Results:**

Trialists frequently expressed views that contradicted the CONSORT (Consolidated Standards of Reporting Trials) guidelines or made inaccurate statements about correct outcome reporting. Common themes were: stating that pre-specification after trial commencement is acceptable; incorrect statements about registries; incorrect statements around the handling of multiple time points; and failure to recognise the need to report changes to pre-specified outcomes in the trial report. We identified additional themes in the approaches taken by researchers when responding to critical correspondence, including the following: *ad hominem* criticism; arguing that trialists should be trusted, rather than follow guidelines for trial reporting; appealing to the existence of a novel category of outcomes whose results need not necessarily be reported; incorrect statements by researchers about their own paper; and statements undermining transparency infrastructure, such as trial registers.

**Conclusions:**

Researchers commonly make incorrect statements about correct trial reporting. There are recurring themes in researchers’ responses when their work is criticised, some of which fall short of the scientific ideal. Research on methodological shortcomings is now common, typically in the form of retrospective cohort studies describing the overall prevalence of a problem. We argue that prospective cohort studies which additionally issue correction letters in real time on each individual flawed study—and then follow-up responses from trialists and journals—are more impactful, more informative for those consuming the studies critiqued, more informative on the causes of shortcomings in research, and a better use of research resources.

**Electronic supplementary material:**

The online version of this article (10.1186/s13063-019-3172-3) contains supplementary material, which is available to authorized users.

## Background

Scientific research commonly falls short of the ideal in both design and reporting, sometimes as a consequence of unavoidable practical issues. Ideally, unavoidable methodological shortcomings should be disclosed in the paper, and additional design and reporting flaws should be discussed in the correspondence after publication, during the process of post-publication peer review. There is extensive anecdotal evidence that this ideal is not met: that methodological shortcomings go undisclosed, that correspondence raising legitimate criticisms is rejected by journal editors, and that engagement by scientists after concerns are raised is not always constructive.

Correct outcome reporting is an important methodological and reporting issue because discrepancies between pre-specified and reported outcomes are a widespread source of bias in clinical trials [1]. Where outcome misreporting is permitted, it increases the likelihood that reported differences have arisen through chance or are exaggerated. Clinical trial registers were established to address selective reporting [2] and require that all pre-specified outcomes be entered at the outset of the trial in a time-stamped and publicly accessible location. Registering clinical trials and pre-specifying their outcomes are mandated by legislation in the US [3] with strong support from the World Health Organization [4], the International Committee of Medical Journal Editors (ICMJE) [2], and an extensive range of professional bodies, funders, ethics committees, publishers, universities and legislatures. The importance of reporting all pre-specified outcomes and documenting changes is also emphasised in the International Conference on Harmonisation of Good Clinical Practice (ICH-GCP) [5] and the CONSORT (Consolidated Standards of Reporting Trials) guidelines [6], which are endorsed by 585 academic journals [7]. However, despite near universal recognition of the importance of this issue and extensive public commitments to address the problem, trial reports in academic journals routinely fail to report pre-specified outcomes, and add in non-pre-specified outcomes, without disclosing that this has occurred. A 2015 systematic review [1] found 27 studies comparing pre-specified outcomes against those reported, in cohorts of between 1 and 198 trials (median *n* = 65 trials). The median proportion of trials with a discrepancy on primary outcomes was 31% (interquartile range 17–45%). Eight studies also assessed the impact of outcome switching on the statistical significance of the published outcome and found that outcome switching favoured the reporting of significant outcomes in half the trials.

In the Centre for Evidence-Based Medicine Outcome Monitoring Project (COMPare), we aimed to explore whether it was possible to publish correction letters on all trials with misreported outcomes in real time, as they were published, in order to ensure that the academic record was more CONSORT-compliant, as per journals’ public commitments. We also aimed to monitor responses from editors and trialists to this standardised set of correction letters, to better understand why outcome misreporting persists despite public commitments to address it; to test the ability of academic journals to self-correct when breaches of their public commitments are reported; and to establish how researchers respond when legitimate objective criticisms of their work are raised.

Here, we analyse the complete set of trialists’ public responses to all published correction letters from COMPare, using thematic analysis to explore inaccurate or problematic statements and misunderstandings around correct outcome reporting. We aimed to answer two related questions. First, what can trialists’ responses to corrections on their own misreported outcomes tell us about trialists’ knowledge of correct outcome reporting? Second, what can a cohort of responses to a standardised correction letter tell us about the techniques that researchers use, explicitly or implicitly, when responding to critical post-publication peer review?

## Methods

Detailed methods of the COMPare trials project are reported in our accompanying paper on the prevalence of misreporting and responses from journals to correction letters [8]. In brief, we monitored for outcome misreporting in five high-impact journals—*New England Journal of Medicine* (NEJM), *Journal of the American Medical Association* (JAMA), *Annals of Internal Medicine*, *British Medical Journal* (BMJ) and *Lancet*—and found a high prevalence of outcome misreporting, consistent with previous work; we therefore submitted 58 correction letters for publication. Twenty-three letters were published: NEJM and JAMA rejected all letters; BMJ accepted all letters as online comments only; *Annals of Internal Medicine* accepted all letters online and two for print; and *The Lancet* accepted the majority of letters in print but with long delays (mean 150 days).

All correspondence published by journals from researchers in response to a correction letter from COMPare was filed, indexed and reviewed by the COMPare team in order to write a reply. Themes from the trialists’ letters were analysed and extracted by the COMPare team and a researcher with expertise in qualitative research methods (CM). Key elements from responses were first extracted systematically to a two-way table with quotes, summaries and further notes explaining the significance of the trialists’ comments as appropriate. These were then organised into the overarching groups, themes and sub-themes presented here.

## Results

The level of engagement with published correction letters was high. Overall, 20 teams of trialists replied to the 23 published letters: two in *Annals*, two in the BMJ, and 16 in *The Lancet*. Nearly all responses contained inaccurate or problematic statements.

### Researchers’ inaccurate statements about outcome reporting

Trialists frequently expressed views that contradicted the CONSORT guidelines or made inaccurate statements around key issues of correct outcome reporting. We grouped these comments and views into five themes:

i). Incorrect statements about when outcomes should be pre-specified.
ii). Failure to recognise the need to report changes to pre-specified outcomes in the trial report.
iii). Incorrect statements about the role and operation of trial registries.
iv). Stating or implying that it was acceptable to have multiple discrepant sets of pre-specified outcomes contemporaneously, by making reference to inaccessible protocols which were claimed to contain different outcomes, discrepant with those in the contemporaneous registry entry.
v). Incorrect statements around how to pre-specify and report when measuring the same outcome at multiple time points.

Since a thorough explanation of the inaccuracies in these statements requires some detail about the individual trial or the wider context of correct outcome reporting, specific illustrative examples are given in Table 1, alongside explanations of the error made. These examples are taken from Additional file 1—the full table of trialists’ inaccurate statements, with rich information on the individual claims and errors made—which we encourage interested readers to examine.

**Table 1:** Researchers’ inaccurate statements about outcome reporting

Theme	Trialists’ response	Issue
Timing of pre-specification		
Stating or implying that pre-specification after trial commencement is acceptable	“The prespecified analysis of PATHWAY-2 precisely followed a detailed statistical analysis plan (SAP) that was published in BMJ Open before data lock and unblinding of data (4), and was provided in full to *The Lancet*, dated and signed before any unblinding or analysis. All primary and secondary endpoints reported in *The Lancet* were listed at ClinicalTrials.gov before data lock and unblinding” (Trial 57, *Lancet*, 02/04/16).	The authors suggest that pre-specification should happen before “data lock and unblinding”. However, CONSORT item 6b requires that trial reports declare and explain “any changes to trial outcomes after the trial commenced, with reasons” in the paper reporting the results of the trial.
Failure to report changes to pre-specified outcomes in paper		
Failure to recognise that post-commencement changes are acceptable but should be declared in the paper reporting the results of the trial	“Length of stay in survivors and days to death (the fifth so-called new endpoint) are components of length of hospital stay, but they were presented separately to prevent bias from higher mortality in either group that resulted in a difference in length of stay between groups” (Trial 27, *Lancet*, 30/01/16).	This change from protocol was not mentioned or explained in the paper. CONSORT item 6b requires that trial reports declare and explain “any changes to trial outcomes after the trial commenced, with reasons” in the paper reporting the results of the trial.
Stating that pre-specified outcomes missing from the trial report, or declarations of changes, will be reported elsewhere but failing to declare this in the trial report	“With regards to cost outcomes, both the primary and four of the eight so-called missing secondary outcomes (therapy costs, quality of life, institutionalisation, and cost-effectiveness ratios) will be presented in a later publication as stated in the headline paper” (Trial 27, *Lancet*, 30/01/16).	Changes from pre-commencement outcomes should be declared in the paper reporting the results, as above. Note that while the authors state the additional outcomes “will be presented in a later publication as stated in the headline paper”, there is no such disclosure in the paper; we asked the authors to identify it in our follow-up letter; this was not published and we received no reply.
Registries		
Incorrect statements about registries	“Trial registries often do not request or have space for sufficient detail about secondary outcomes” (Trial 10, *Lancet*, 16/04/16)	There are no restrictions on posting secondary outcomes to registers.
Multiple sets of discrepant pre-specified outcomes		
Making reference to protocols that are publicly inaccessible, or were published after trial commencement, which allegedly contain outcomes that are discrepant with registry entries but consistent with the published report	“The trial was registered at ClinicalTrials.gov where we indicated that the primary outcome was... The protocol was also sent to *The Lancet* shortly after the study started and a summary of the protocol was published. The submitted protocol clearly indicated that the primary outcome was… all outcomes at 2 years prespecified in the submitted protocol were reported but were not included in the published protocol” (Trial 10, *Annals*, 16/04/16).	The argument appears to be that there is a publicly inaccessible pre-commencement protocol that contains pre-specified outcomes different from those in the contemporaneous pre-commencement registry entry. There is no methodological justification for discrepant outcomes between registry entry and protocol for the same trial at the same time point: the two should be the same, and changes after trial commencement should be discussed in the results paper. Registries were devised as a publicly accessible location for trial information specifically to prevent selective outcome reporting. Having multiple discrepant sets of pre-specified outcomes, with the option to choose between multiple discrepant documents, undermines the purpose of pre-specifying outcomes.
Making reference to multiple discrepant sets of pre-specified outcomes	“All primary and secondary endpoints reported in *The Lancet* were listed at ClinicalTrials.gov before data lock and unblinding. The protocol… also posted on the public domain, EudraCT, before patient recruitment, correctly identified the primary objective… The primary outcome measure was correctly stated on EudraCT” and so on (Trial 57, *Lancet*, 02/04/16).	This trial had multiple different sets of conflicting “prespecified” outcomes in different locations at similar dates. For example, different outcomes are registered on ClinicalTrials.gov in February and July 2015, and both sets of outcomes in turn are inconsistent with those in the protocol of June 2015.
Issues with time points		
Incorrect statements around issue of multiple time points	“We do not see how these multiple measurement time points should be counted as separate outcomes, as the procedure of the COMPare team seems to propose. We think this leads to misuse of overall statistics on their website and exaggerated conclusions about the magnitude of outcome switching in RCTs” (Trial 70, BMJ, 04/02/16).	The trial report states “Secondary outcome measure were symptoms of depression and anxiety measured with the CES-D and HADS-A at baseline and at 3, 6, 9, 12, 18 and 24 months”; and all of these time points are then separately reported in Table 5 of the trial report as a mean with a standard deviation. These are all outcomes, according to the CONSORT guidelines. None of these time points was pre-specified before trial commencement; therefore, 21 non-pre-specified secondary outcomes were reported.

References throughout are to the correspondence archive at http://COMPare-trials.org/data containing the full public correspondence on all trials, and all correspondence with editors, organised by trial ID and date, or journal name for general correspondence. Abbreviations: *BMJ British Medical Journal*, *CES-D* Center for Epidemiologic Studies - Depression, *COMPare* Centre for Evidence-Based Medicine Outcome Monitoring Project, *CONSORT* Consolidated Standards of Reporting Trials, *HADS-A* Hospital Anxiety and Depression Scale - Anxiety, *PATHWAY* Prevention And Treatment of Hypertension With Algorithm-based therapy, *RCT* randomised controlled trial

### Researchers’ response styles

The second group of themes relates to the more general techniques and approaches used by researchers when responding to criticism of their work, whether consciously or unconsciously. We identify this group of themes broadly as “rhetoric”, although we do not suggest that this was always the explicit intention of the researchers: we explore this further in the Discussion section. We identified five core themes in this group, as set out in Table 2.

**Table 2:** Themes and sub-themes in “researchers’ response styles”

Researchers’ response styles	
*Diversion*	
1. Stating that trials are hard work to conduct	
2. Stating that other issues are more important	
3. Response based on issues not raised by COMPare	
4. *Ad hominem*	
*Challenging legitimacy of discussion*	
1. Expressing a preference for conventional peer review over open post-publication critical appraisal	
2. Disagreement with the general approach of COMPare/CONSORT	
3. Asserting that there should be the opportunity to post comments on COMPare’s own raw data sheets online	
4. Stating that they applaud the overall goal, followed by a caveat	
Trust	
1. Statement that discrepancies were not motivated by desire to manipulate findings	
2. Stating that outcome misreporting doesn’t matter if the main results of the study are unlikely to be affected	
*Incorrect statements about outcome reporting in their own paper*	
1. Denying that specific misreported outcomes were indeed misreported	
2. General denial of COMPare’s findings	
*Technical/Rhetorical*	
1. Appealing to the existence of a novel category of outcomes whose results need not be correctly reported	
2. Stating that space constraints prevent all pre-specified outcomes from being reported	
3. Stating that it is not necessary to pre-specify some outcomes as they are “necessarily implied” by other outcomes	
4. Inaccurate statements about COMPare’s methods	

Abbreviations: *COMPare* Centre for Evidence-Based Medicine Outcome Monitoring Project, *CONSORT* Consolidated Standards of Reporting Trials

Under “*diversion*”, we included responses that we regarded as distractions from a focused discussion on correct outcome reporting. These included statements that trials are hard work to conduct, stating that other issues are more important, and responses based on issues that were not raised by COMPare. We also include *ad hominem* comments.

Under “*challenging legitimacy of discussion*”, we included responses that we regarded as challenging whether an open conversation about CONSORT-compliant outcome reporting should happen at all. These included the following: expressing a preference for conventional peer review over open post-publication critical appraisal; disagreement with the general approach of COMPare/CONSORT; asserting that there should be the opportunity to post comments on COMPare’s own raw data sheets online; and stating that they applaud the overall goal of COMPare, followed by a caveat. Examples here include author responses criticising COMPare for focusing on “the negative”, such as “Although we commend the efforts of COMPare, we find it difficult to appreciate their focus on the negative aspects of published studies” (Trial 70, BMJ, 04/02/16).

Under “*Trust the trialist*”, we included responses asserting that trialists should be relied upon to make unbiased judgements about whether to report a finding. This included statements that discrepancies were not motivated by desire to manipulate findings and statements that outcome misreporting does not matter if the main results of the study are unlikely to be affected.

Under “*Incorrect statements about outcome reporting in their own paper*”, we included individual responses denying that specific misreported outcomes were indeed misreported and general denial of COMPare’s findings. We do not suggest that any or all of these examples are deliberate misrepresentations: however, we categorised them in the “response styles” group because these were researchers making incorrect statements about the factual content of their own specific publication, rather than technical misunderstandings of what constitutes correct outcome reporting in general.

Lastly, some themes within the “responses styles” group appealed to specific technical issues but overall appeared to us to be used in a way that was more strategic or rhetorical than those within the first group of statements, which were clearly factually inaccurate. We characterised these as “*technical - rhetorical*”. These included appealing to the existence of a novel category of outcomes whose results need not be correctly reported, stating that space constraints prevent all pre-specified outcomes being reported, stating that it is not necessary to pre-specify some outcomes as they are “necessarily implied” by other outcomes, and making inaccurate statements about COMPare’s methods. The full table of examples grouped by theme is in Additional file 2; a selection is presented in Table 3.

**Table 3:** Researchers’ response styles

Theme	Trialists’ response	Issue
Diversion		
Stating that trials are hard work to conduct	“Our 13 authors and 44 collaborators dedicated almost a decade to bringing to fruition the first prospective comparison of drug treatments for resistant hypertension”. “The obstacles to performing all clinical trials these days are immense” (Trial 57, *Lancet*, 02/04/16).	
Stating that other issues are more important	“We also believe that larger issues are at stake in keeping control over the procedure of a pragmatic trial that merit more discussion on its influence than outcome counting, e.g. the development and implementation of interventions, training professionals to comply with strict protocols, setting up a trial in multiple centres using the same procedures, keeping contact with participants to avoid drop-out (often impossible to avoid due to illness or death), blinding of outcome assessors, medical ethics, phishing incidents [2], etcetera” (Trial 70, BMJ, 04/01/16).	
Response based on issues not raised by COMPare	“The only deviation we can see from the ISCRTN entry is the fact that we exceeded our initial trial sample size (691 in the published report versus 600 in the trial registry). We don’t think this is a hanging offence, and we did this to ensure we maintained our level of pre-specified statistical power when follow up was a little lower than we anticipated (such things do happen). We note that trials commonly fail to achieve their pre-specified sample size ...” (Trial 47, BMJ, 21/12/15).	All examples given here discuss issues that COMPare did not raise. None of them justifies undeclared discrepancies between pre-specified and reported outcomes. For trial 47, for example, the only publicly accessible pre-commencement outcomes were in the ISCRTN registry entry. This contains 11 pre-specified secondary outcomes, three of which are not reported in the BMJ paper, with no declaration of their omission.
*Ad hominem* comments	“In the last few months, the COMPare team has monitored five top journals to analyse trials on outcome switching. Based on their interpretation of the CONSORT guidelines, comments on outcome switching have been produced. However, until now, their work has not gained or secured widespread support - neither by funders (their project is paid out-of-pocket) nor by the editors of the five top journals who do not seem keen to publish their comments...” (Trial 70, BMJ, 01/04/16). “With their approach of criticising and not being open to discussion... COMPare places themselves outside the research community. Although it can be debated to what extent it is possible to develop and criticise an aspect of science from the outside by persons not directly involved [4], we believe the research community should be critical, but with the aim to support and improve science” (Trial 70, BMJ, 01/04/16).	
Challenging legitimacy of discussion		
Expressing a preference for conventional peer review over open post-publication critical appraisal	“In retrospect, we believe that expert and constructive peer reviews are sufficient to raise science to a higher level” (Trial 70, BMJ, 04/01/16).	
Disagreement with the general approach of COMPare	“The COMPare team might well catch some true outcome switching and ‘fishing’; however, in their net they are also catching researchers who have not switched outcomes or selectively reported, but have simply made minor errors of omission in their registry entries” (Trial 10, *Lancet*, 23/07/16).	
Asserting that there should be the opportunity to post comments on COMPare’s own raw data sheets online	“We hope the COMPare project team will take into account our comments, post our response on their website ...” (Trial 17, *Lancet*, 14/05/16).	We set out to correct the record of misreported trials in the journal where they were misreported. Although we shared our raw underlying data sheets in an online repository, we felt that the appropriate place for a critical discussion about the correct reporting of the pre-specified outcomes was the journal where the trial results were reported. Consigning the discussion to our online data repository, rather than journal correspondence, would significantly reduce the visibility of a constructive discussion around correct outcome reporting.
Stating that they applaud the overall goal, followed by a caveat	“While we support the principles of COMPare ...” (Trial 25, *Annals*, 11/12/15).	
“Trust the trialist”		
Statement that discrepancies were not motivated by desire to manipulate findings	“In response to Dale and colleagues, it should be noted that the PATHWAY programme was devised by eight academic investigators with no vested interests other than a wish to answer previously intractable questions arising from centuries of cumulative experience of hypertension practice and trials” (Trial 57, *Lancet*, 02/04/16).	It is unlikely that all outcome misreporting reflects a deliberate attempt by trialists to misrepresent a study’s findings; however, a culture of permissiveness around correct outcome reporting does permit misrepresentation.
Stating that outcome misreporting doesn’t matter if the main results of the study are unlikely to be affected	“If Dale and colleagues’ inference is that spironolactone’s overwhelming superiority over licensed antihypertensive drugs is due to selection of multiple results” (Trial 57, *Lancet*, 02/04/16).	It is unlikely that all outcome misreporting changes or exaggerates the overall finding from a trial. However, the evidence from the current systematic review shows that this tends to be the case, and a culture of permissiveness around correct outcome reporting facilitates such misrepresentation.
Incorrect statements about outcome reporting in their own paper		
Denying that specific misreported outcomes were indeed misreported	“We have clarified in the Methods section that physician diagnosed pneumonia was not a primary outcome” (Trial 27, *Lancet*, 30/01/16).	COMPare searched the paper repeatedly and found no such disclosure; in fact, the paper in question explicitly describes physician-diagnosed pneumonia as the “co-primary outcome”.
General denial of COMPare’s findings	“We whole heartedly agree with the scrutiny of endpoints in high-profile clinical trials such as ours that Dale and colleagues have performed. It is reassuring that this analysis indicates that our Article is correctly reported and as such is consistent with the scientific and clinical intent of the trial as described in the protocol” (Trial 56, *Lancet*, 11/06/16).	This trial was not correctly reported, as explained in the COMPare letter to which this comment was a reply: two pre-specified outcomes were unreported, and four additional outcomes were reported without disclosing that they were novel.
Technical/Rhetorical		
Appealing to the existence of a novel category of outcomes whose results need not be correctly reported	“None of these are key secondary endpoints” (Trial 56, *Lancet*, 11/06/16).	The outcomes pre-specified in the registry entry were not reported for this trial. The phrase “key secondary outcomes” is one used by the WHO, in their list of 20 items that should be in all registry entries, to denote all the secondary outcomes pre-specified in the registry, which should all be reported.
Stating that space constraints prevent all pre-specified outcomes from being reported	“As indicated by Aaron Dale and colleagues, two of three pre-specified primary outcomes were not fully described in the results section of our Article for word limitation reasons” (Trial 29, *Lancet*, 11/06/16).	While the authors appeal to length limits, this paper reported an additional outcome (“distribution of clinical stages of cancer”), stratified by clinical stage, percentage of reported breast cancer–positive patients and relative sensitivity. This resulted in their reporting 16 additional outcomes that were not pre-specified (none of which was declared as non-pre-specified). Reporting non-pre-specified outcomes was common throughout the project.
Stating that it is not necessary to pre-specify some outcomes as they are “necessarily implied” by other outcomes	“the adjudication of the pre-specified endpoints of any myocardial infarction, target vessel myocardial infarction, revascularisation, or target vessel revascularisation, necessarily implies the assessment of the non-target vessel myocardial infarction and the non-target vessel revascularisation” (Trial 17, *Lancet*, 14/05/16).	This is an additional outcome that was not pre-specified. Clear pre-specification is required by registers, regulators, and CONSORT in order to avoid selective reporting. Unnecessary flexibility leaves trialists the option to selectively report outcomes, with no public record of the original intentions of the trial.
Inaccurate statements about COMPare’s methods	“... we suggest that a trial’s published protocol should also be reviewed by COMPare in tandem with its Registry entry as part of their process” (Trial 25, *Annals*, 11/12/15).	The COMPare method used both: protocols were used preferentially; if these were unavailable, or published after trial commencement, then the trial registry entries were used instead.

References throughout are to the correspondence archive at http://COMPare-trials.org/data containing the full public correspondence on all trials, and all correspondence with editors, organised by trial ID and date, or journal name for general correspondence. Abbreviations: *BMJ British Medical Journal*, *COMPare* Centre for Evidence-Based Medicine Outcome Monitoring Project, *CONSORT* Consolidated Standards of Reporting Trials, *PATHWAY* Prevention And Treatment of Hypertension With Algorithm-based therapy, *WHO* World Health Organization

### Researchers’ correction of errors

Only eight teams of trialists clearly and publicly acknowledged a specific discrepancy between their pre-specified and reported outcomes, out of 58 misreported trials. Of these, all but two acknowledgements of error were given in the context of caveats or further problematic statements about correct outcome reporting. Only one misreported trial was updated with a correction. In addition, three teams of trialists incorrectly stated that they should have retrospectively updated the pre-specified outcomes in the registry to ensure that reported outcomes were consistent with those pre-specified and presented this as an acknowledgement of an error; in other words, while they acknowledged that an error was made, they seemed to continue to misunderstand the nature of the error. Examples are given in Table 4, taken from the full sample in Additional file 3.

**Table 4:** Researchers’ correction of errors

Theme	Trialists’ response	Issue
Acknowledgement of error by trialists		
Clear acknowledgement of CONSORT breach and then a clarification	“One prespecified secondary outcome from the protocol (assisted vaginal delivery) was omitted from the analysis plan in error, and, therefore, not reported” (and further corrections for same trial) (Trial 46, *Lancet*, 14/04/16).	We regard a clear correction as best practice. In our cohort of 58 submitted letters, it was uncommon.
	“We did not report the results of the Steatotest as we had incomplete data for this because of sample haemolysis… For the purpose of transparency, we include the median values at baseline…” “We presented data on three parameters that had not been predefined as secondary endpoints…” (Trial 56, *Lancet*, 11/06/16).	Note that these trialists also introduced a spurious distinction regarding unreported outcomes (“none of these are key secondary endpoints”).
	“We accept that our reporting of the change in the primary depression outcome in the BMJ paper could have been better ... we accept that, by rule, we have failed to be entirely transparent and we meet their criteria for such a rating” (Trial 47, BMJ, 14/01/16).	
Correcting the wrong error	“We have reviewed these discrepancies and concede that we failed to fully update the trial registry” (Trial 8, *Lancet*, 13/02/16).	The error was not failure to update the registry entry but rather failure to report pre-specified outcomes or document discrepancies.

References throughout are to the correspondence archive at http://COMPare-trials.org/data containing the full public correspondence on all trials, and all correspondence with editors, organised by trial ID and date, or journal name for general correspondence. Abbreviations: *BMJ British Medical Journal*, *CONSORT* Consolidated Standards of Reporting Trials

### Correcting inaccurate statements in researchers’ responses

COMPare submitted letters for publication setting out corrections and clarifications to all errors and inaccurate statements noted in Tables 1 and 3. To date, only two of these letters have been published in *The Lancet*, and none in print by either the BMJ or *Annals*: both BMJ and *Annals* accepted comments online (*Annals* only if brief); NEJM and JAMA rejected all initial correspondence notifying readers and researchers of outcome misreporting, as previously reported; therefore, no interaction with these trialists was possible.

## Discussion

### Summary

We found that trialists engage at length with published correspondence identifying misreporting of pre-specified outcomes. However, inaccurate statements and misunderstandings about what constitutes correct outcome reporting were common, even among trialists publishing in high-impact journals. In addition, response styles such as *ad hominem* criticism, distraction and denial were commonly used.

### Strengths and weaknesses

A larger sample of trials and trialists would have been preferable. Our study included the full correspondence with 20 teams of researchers and could have included all 58 trials with misreported outcomes identified during COMPare: however, our ability to engage with trialists was hindered by journal editors rejecting the majority of initial correction letters identifying misreporting of outcomes, despite clear evidence that these trial reports had all breached the CONSORT guidelines on correct outcome reporting; and by journals rejecting the majority of COMPare follow-up letters engaging with errors in trialists’ responses, as discussed below.

### Context of other research

There have been extensive previous anecdotal reports in the grey and academic literature of researchers’ failures to engage constructively with post-publication peer review that is critical of study methods and results. COMPare is the first study to approach and document this problem systematically with a standardised set of correction letters and on an objective issue of accurate study reporting in line with standard best practice guidelines. COMPare is also the first study to systematically solicit and analyse detailed technical responses from a representative sample of trialists and engage them in a practical real-world detailed discussion of outcome reporting using examples of misreporting from their own work to identify knowledge gaps. There has been extensive previous research establishing the high prevalence of outcome misreporting [1] and other reporting flaws [9] and some questionnaire data on the limitations of trialists’ knowledge around correct outcome reporting. One previous survey on the prevalence of outcome misreporting also engaged trialists in semi-structured telephone interviews to explore their reasons for not reporting specific outcomes: this study design yielded less detail in terms of specific misunderstandings or inaccurate statements than ours; however, consistent with our findings, they did report that trialists “seemed generally unaware of the implications for the evidence base of not reporting all outcomes and protocol changes” and that some regarded non-significant results as “uninteresting” [10]. Another series of semi-structured telephone interviews with 59 trialists similarly yielded the finding that non-significant findings are sometimes regarded as uninteresting, and space constraints may hinder complete outcome reporting [11].

### Interpretation

It is challenging to reach a fair interpretation of what drives trialists’ incorrect statements about correct outcome reporting. To retain neutrality, we have labelled all of these statements as “inaccurate” rather than either “misunderstandings” or “misleading comments” because it is not possible to know the level of knowledge for all researchers assessed. Some, none, or all of the inaccurate statements documented may have represented genuine misunderstandings or a lack of knowledge. To expand on this, it is possible that these trialists do not know what correct outcome reporting consistent with CONSORT looks like and are making genuine unintended errors; it is also possible that they do not care about CONSORT and are speaking implicitly or explicitly to a more vague alternative set of unstated principles around correct outcome reporting which they regard as superior.

Equally, some, none, or all of the inaccurate statements may have been used deliberately in an attempt to deflect criticism and publicly defend what the researchers knew to be misreporting. This would imply that researchers were not primarily concerned with what constitutes correct outcome reporting but rather with defending their reputation. At face value, it seems likely that anyone with good knowledge of correct outcome reporting, and concerned to defend their reputation, would be equally concerned by the negative reputational consequences of formally publishing a letter that contained clear misunderstandings around what constitutes correct outcome reporting. For this to be a rational position therefore, researchers would also have to believe that the public discussion is likely to be brief, poorly understood by onlookers (or ignored), and unlikely to lead to a resolution establishing who was right or wrong on matters of fact.

To an extent, this view is vindicated by the initial findings of COMPare, where journal editors mostly rejected letters reporting outcome misreporting, and often defended such misreporting, despite the journal’s being publicly listed as endorsing CONSORT. Researchers may also feel bolstered by the fact that a journal has published their paper after peer review and is therefore likely to feel some commitment to supporting it; by the fact that a paper with misreported outcomes is unlikely to be retracted, or even corrected, so this is just a matter for correspondence; and by the fact that letters in journals have lower visibility than original research. Related to the issue of managing the visibility of correspondence, it is notable that some research teams suggested that the discussion on their misreported outcomes should take place as annotations to our raw data archive rather than in the journal where their research was published.

There is also a third option combining both of the previous two: that these were “motivated misunderstandings”, where researchers do not have a full clear working understanding of correct outcome reporting, but are not inclined to develop one, and merely seek to survive a single round of public criticism in the reasonable expectation that any potentially inaccurate statements will not be exposed in the full cycle of post-publication peer review. Under any of these three models, two core problems obtain. First, the failure of journals to curate post-publication peer review such that errors on matters of fact are resolved has resulted in a sub-optimal approach from scientists to the accurate reporting of their own work; second, a widespread lack of knowledge around correct outcome reporting has contributed to both misreporting and poor discourse around that misreporting.

Separately to this, we found many examples of obfuscation, *ad hominem* criticisms, and other techniques that can fairly be described as “rhetorical”. Although these do not directly relate to the specific issues of outcome reporting and may not be reasonably regarded as unacceptable *per se*, they are part of a broader set of processes restricting adequate scrutiny of correct reporting. It is also worth noting that we may not have had access to the full breadth of *ad hominem* comments, because we do not have access to the text of the letters submitted, only those published. Letters published in *The Lancet* (the majority in our cohort) go through an extensive process of editorial control, proof-reading, and some re-drafting; we note that the tone of BMJ “rapid responses”—which are posted online within hours of submission, and usually unchanged—was often much more raw than the formal letters published after a delay in *The Lancet*. On the issue of self-censorship, it is also possible that the constitution of the COMPare team reduced the quantity of *ad hominem* criticism. Because such criticism is based on denigrating the recipient rather than ideas, it likely to be mediated by perceived relative social status, which in turn is mediated by factors such as class, gender and race. It is therefore possible that we received less than a different team might have done, since those submitting correction letters were all academics at Oxford, recently listed as the leading medical research institute in the world; we have a professor and other senior staff on our team; and the COMPare correspondents named on correction letters were all male and mostly identifiable as White British.

A related issue of power relations concerns the question of who should decide whether an outcome requires reporting. CONSORT is clear that all pre-specified outcomes should be reported or discrepancies flagged. As per our section “Trust the trialist”, many trialists stated that outcome switching is irrelevant if it does not affect the outcomes of the study. Ultimately, in our view, this reflects scientists asserting that they should be trusted to faithfully report summary results without oversight and asserting authority over the data as if it were owned by the trialist rather than participants or the wider community. This is inconsistent with the wider societal shift towards greater transparency and accountability in science.

### Implications

We identify various implications of our study for editors, funders, trial registries, and ethics and regulators; for initiatives seeking to improve research methods and reporting; and for researchers whether they are publishing work, responding to published work, or consuming published work. We have found that trialists publishing in high-impact journals routinely misreport their pre-specified outcomes and, when challenged, regularly make incorrect statements on the topic of correct outcome reporting. This may reflect a lack of knowledge: where this is the case, we suggest that better education and training on research methods may improve matters. However, trialists are also deprived by journal editors of important feedback that would likely help to raise standards. Journals could improve standards by policing correct outcome reporting, giving feedback to trialists where they have submitted papers that fail to comply with CONSORT standards on outcome reporting, and encouraging trialists to engage positively with feedback on methodological and reporting flaws, as already recommended in ICMJE guidance. In some cases, the incorrect statements made by trialists may reflect deliberate or unconscious use of superficially plausible but incorrect arguments as a rhetorical device to defend misreported studies. Where this is the case, research integrity training may improve standards, alongside support for ongoing efforts to foster a culture of positive and reciprocal critical appraisal in scientific discourse.

Trial registries should emphasise that information on registries is important, give additional guidance on the specific elements required, and give feedback to trialists when registry entries fall short on required information. Registry managers and ethics committees could remind trialists that pre-specified outcomes in protocols and registry entries should match. Ethics committees and funders could take responsibility for “closing the loop” with a report at the end of a project, confirming that all results have been appropriately published, deviations from the ethically approved protocol accounted for, and post-publication peer review engaged with constructively. Organisations such as the EQUATOR (Enhancing the Quality and Transparency of Health Research) network, running the CONSORT guidelines, should disambiguate any areas in their recommendations that are perceived by researchers as unclear, and could offer a service for trialists or journals to check that trials have been correctly reported across a range of methodological issues. Lastly, consumers of the research literature should be aware that the peer-reviewed academic literature contains a high prevalence of misreported research and that efforts to correct this are routinely resisted by journal editors. The majority of initial letters from COMPare were rejected, and the overwhelming majority of responses to authors’ responses were also rejected. Therefore, the extensive errors documented in Table 1, in Additional file 1, and in the longer COMPare correspondence archive currently stand unaddressed and without a published response in the scientific literature, other than in this article.

Lastly, we believe that the rhetorical approaches demonstrated by many respondents in our cohort—such as diversion, hostility, and challenging the legitimacy of having a discussion—will be recognised by academics more broadly. We hope that this will be useful for those writing letters criticising the content of a scientific paper or anxious about a response they have received from an author. Although clarity and professionalism are important, the wide variation in responses we received to our large set of identical correction letters strongly suggests that hostile or obfuscatory responses are, at least in part, a function of the responding authors rather than the letter that stimulated the response.

### Future research

The academic literature already contains a very large number of studies which retrospectively document the overall prevalence of methodological flaws or reporting discrepancies in clinical trials. These studies are expensive, requiring skilled labour from experienced researchers to identify a large number of flaws in published research. In our view, by publishing these findings as only a single anonymised prevalence figure, these teams are failing to maximise the value and impact of their work. We suggest that wherever research is done documenting the prevalence of flaws in individual studies, researchers should also submit letters for publication on each individual paper where a shortcoming has been identified, in order to alert other consumers of the academic literature to the presence of specific flaws in specific studies, to generate informative or corrective discussion with the researchers concerned, to raise awareness among individual researchers about flaws in their own research, and to generate dialogue allowing methodologists to better understand the misunderstandings or structural challenges driving methodological and reporting flaws, and so devise interventions to improve standards.

## Conclusions

Outcome misreporting is common in clinical trials. Journal editors and trialists do not engage constructively when misreporting is reported. It is unlikely that these problems are limited to the specific issue of outcome reporting in the specific field of clinical trials research. The findings here, and in our accompanying article on COMPare, provide strong evidence from a large cohort of studies that the institutions of research in practice commonly fall short of the scientific ideal.

## Additional files

Additional file 1: Full table of examples of trialists’ inaccurate statements. References are to correspondence archive at http://COMPare-trials.org/data. (PDF 110 kb)

Additional file 2: Full table of examples of trialists’ response styles. References are to correspondence archive at http://COMPare-trials.org/data. (PDF 93 kb)

Additional file 3: Full table of examples of trialists’ error acknowledgements. References are to correspondence archive at http://COMPare-trials.org/data. (PDF 84 kb)

## Abbreviations

BMJ: *British Medical Journal*
COMPare: Centre for Evidence-Based Medicine Outcome Monitoring Project
CONSORT: Consolidated Standards of Reporting Trials
ICMJE: International Committee of Medical Journal Editors
JAMA: *Journal of the American Medical Association*
NEJM: *New England Journal of Medicine*
