# Supplementary material for: COMPare: Qualitative analysis of researchers’ responses to critical correspondence on a cohort of 58 misreported trials
Source: Trials. 2019 Feb 14;20:124. doi: 10.1186/s13063-019-3172-3 (PMC6374909; doi:10.1186/s13063-019-3172-3)
Supplement: Supplementary file 1 — Full table of examples of trialists’ inaccurate statements. References are to correspondence archive at http://COMPare-trials.org/data. (PDF 110 kb) [file 13063_2019_3172_MOESM1_ESM.pdf]

Theme	Trialists' response	Issue
**Timing of prespecification**		
Stating or implying that prespecification after trial commencement is acceptable.	"The prespecified analysis of PATHWAY-2 precisely followed a detailed statistical analysis plan (SAP) that was published in BMJ Open before data lock and unblinding of data (4), and was provided in full to The Lancet, dated and signed before any unblinding or analysis. All primary and secondary endpoints reported in The Lancet were listed at ClinicalTrials.gov before data lock and unblinding." (Trial 57, Lancet, 02/04/16).	The authors suggest that pre-specification should happen before "data lock and unblinding". However, CONSORT item 6b requires that trial reports should declare and explain "any changes to trial outcomes after the trial commenced, with reasons" in the paper reporting the results of the trial.
	""[NIHSS] score, change in NIHSS score at 14 days, mortality at 14 days, and modified Rankin Scale 0–2 at 90 days [were prespecified]" (Trial 27, Lancet, 30/01/16)	These appear only in the modified 2014 registry entry (the trial began in 2008). None appear anywhere in the original registry entry, nor in a pre-commencement protocol.
	"All the primary and secondary endpoints in the Article were the same as those in the protocol." (Trial 9, Lancet, 23/01/16)	Trialists state the reported outcomes reflect the 2011 protocol: this document is marked as last updated in February 2015, with no record of amendments, three years after the start of the trial.
	"There is no inconsistency between our trial report and our pre-specified trial protocol which has been in the public domain for several years." (Trial 47, BMJ, 21/12/15)	The protocol they cite as "pre-specified" is dated 10th December 2012, however data collection for the trial started in December 2009, 3 years earlier. We therefore used the ISRCTN registry entry that predates the start of the trial to establish the pre-specified outcomes.
	"Full details of the pre-specified outcomes of our randomized controlled trial are provided in the trial protocol, which was published at the start of the trial in an open accessed journal." (Trial 70, BMJ, 04/02/16)	The protocol they describe as "published at the start of the trial" was received for publication in July 2013, and published August 2013; data collection began in 2012.
	"The rationale and design of the EXAMINATION trial,1 reported the primary and the secondary endpoints to be assessed at 1 year 2 and every year up to 5 year follow-up." (Trial 17, Lancet, 14/05/16)	Every new timepoint is a new outcome. The "rationale and design" publication cited by the authors [4] was published in December 2011, 3 years after trial commencement in December 2008 [5]. This document can therefore not be said to contain "prespecified" outcomes.
	"During the study, a protocol amendment was made in which the study duration was extended from 12 months to 24 months. The statistical analysis plan was subsequently amended and completed before database locking." (Trial 26, Lancet, 20/02/16)	Changes after commencement should be reported in the paper according to CONSORT; these changes were not.
	"The analysis plan used in the paper provides a comprehensive list of primary and secondary outcomes. The plan was completed and published before data lock and unblinding of treatment allocation." (Trial 46, Lancet, 14/05/16)	Changes after commencement should be reported in the paper according to CONSORT; these changes were not.
**Failure to report changes to prespecified outcomes in paper**		
Faiiure to recognise that post-commencement changes are acceptable, but should be declared in the paper reporting the results of the trial.	"Length of stay in survivors and days to death (the fifth so-called new endpoint) are components of length of hospital stay, but they were presented separately to prevent bias from higher mortality in either group that resulted in a difference in length of stay between groups." (Trial 27, Lancet, 30/01/16)	This change from protocol was not mentioned or explained in the paper. CONSORT item 6b requires that trial reports should declare and explain "any changes to trial outcomes after the trial commenced, with reasons" in the paper reporting the results of the trial.
	"These small changes were made at the suggestion of the data monitoring and trial steering committees, amended in the trial protocol, and approved by the Research Ethics Committee. The timeline and justification for these changes are fully documented in the full published NIHR Health Technology Assessment report." (Trial 8, Lancet, 13/02/16)	Changes to prespecified outcomes should be reported in the paper or papers reporting the results of the trial. Note that the NIHR HTA report mentioned in the authors' response was neither cited nor mentioned in the journal paper reporting the results of the trial.

Theme	Trialists' response	Issue
	"trials should not be set in stone. When legitimate reasons exist to alter the protocol or generate new data, they are agreed by the trial steering committee and the funder, and then reported in the paper as changes, and the reader can judge for themselves whether the spirit of the original protocol has been maintained. The process of sensible, agreed, and documented modification of trial protocols allows us to have trials that uphold the original idea but remain fit for purpose." (Trial 7, Lancet, 23/01/16)	None of the changes made during the course of the trial are documented in the report of the trial. CONSORT item 6b requires that trial reports should declare and explain "any changes to trial outcomes after the trial commenced, with reasons" in the paper reporting the results of the trial.
	"There was complete transparency in relation to protocol changes in our full report (freely available in the public domain) where we devoted a whole chapter to the subject." (Trial 47, BMJ, 14/01/16)	CONSORT item 6b requires that trial reports should declare and explain "any changes to trial outcomes after the trial commenced, with reasons" in the paper reporting the results of the trial. This was not done.
	"We chose to omit SVR4 results because the size and complexity of the study (14 treatment groups with patients of all genotypes) forced us to be selective about the data we could include, and SVR4 is a measure of very limited interest to clinicians in the era of direct-acting antivirals" (Trial 45, Annals, 02/03/15)	
Stating that prespecified outcomes missing from the trial report, or declarations of changes, will be reported elsewhere, but failing to declare this in the trial report.	"With regards to cost outcomes, both the primary and four of the eight so-called missing secondary outcomes (therapy costs, quality of life, institutionalisation, and cost-effectiveness ratios) will be presented in a later publication as stated in the headline paper." (Trial 27, Lancet, 30/01/16)	Changes from pre-commencement outcomes should be declared in the paper reporting the results, as above. Note that while the authors state the additional outcomes "will be presented in a later publication as stated in the headline paper" there is no such disclosure in the paper; we asked the authors to identify it in our follow-up letter, this was not published and we received no reply.
	Of the 17 secondary pre-specified outcome measures, all of them have been or will be reported in the primary paper or in secondary papers that are currently being prepared. These papers will report on the as yet unpublished outcomes. (Trial 25, Annals, 11/12/15)	Changes from pre-commencement outcomes should be declared. Furthermore the manuscript for this trial reads: "Dr. MacPherson (the manuscript's guarantor) affirms that ... any discrepancies from the study as planned (and, if relevant, registered) have been explained." There were discrepancies, for the majority of prespecified outcomes, and none were explained.
	"Far from seeking to omit this secondary outcome, we currently have a paper in preparation to report the economic dimension of cCBT. Should the BMJ wish to publish this, we will be happy to oblige." (Trial 47, BMJ, 21/12/15)	Changes from pre-commencement outcomes should be declared.
	"The timeline and justification for these changes are fully documented in the full published NIHR Health Technology Assessment report." (Trial 8, Lancet, 13/02/16)	
	"...results are publicly available on ClinicalTrials.gov (number NCT01520909) and the GlaxoSmithKline clinical study registry (number 115450)." (Trial 9, Lancet, 23/01/16)	
	"We did not report the five pharmacokinetic endpoints in our Article1 but will include them in a separate publication, in which these data will be combined with similar data from the phase 2 PETIT study." (Trial 9, Lancet, 23/01/16)	
	"Most secondary outcomes missing from the paper are data collected for the longer term maternal satisfaction measures and health economic analyses, which will be reported in due course." (Trial 46, Lancet, 14/05/16)	
**Registries**		

Theme	Trialists' response	Issue
Incorrect statements about registries.	"trial registries often do not request or have space for sufficient detail about secondary outcomes" (Trial 10, Lancet, 16/04/16)	There are no restrictions on posting secondary outcomes to registers, indeed this is required by law in the EU and US.
	"The trial registry entry (ClinicalTrials.gov, number NCT01237119) only allows for a limited description of the protocol, hence publication of the full protocol, which contains all the relevant details." (Trial 56, Lancet, 11/06/16)	The researchers imply that the protocol simply contains more detail than the registry entry. However, for this trial the outcomes in the protocol were simply different outcomes to those in the registry. A trial registry entry cannot contain all the information that would appear in more comprehensive protocols; but we are not aware of word-length restrictions on clinical trial registries that would prevent outcomes being adequately prespecified.
	"While we support the CONSORT guidelines on best practice in trial reporting, we are concerned about the ambiguity of Registry entry labelling and scope for misinterpretation either by researchers when entering data at the outset of a trial, or by commentators, as is the case we discuss here." (Trial 25, Annals, 11/12/15)	Trialists are responsible for ensuring that information they enter for their own trial registry entries and protocols is not "ambiguous".
	"We thank Drs Drysdale, Slade, and Goldacre for their interest in our paper. Their letter refers to the study objectives that were originally posted on the clinicaltrials.gov when the trial was registered on May 20, 2013 (NCT01858766). However, the administrators of the website subsequently requested that instead of study objectives we provide specific efficacy and safety endpoints, which they posted on November 7, 2013." (Trial 45, Annals, 02/03/16)	The outcomes were changed during the trial, but this was not disclosed. To explain this, the trialists seek to draw a distinction between "objectives" initially registered and "endpoints" registered later. The original prespecified outcomes, described as "objectives" rather than "endpoints", were: SVR12, safety and tolerability at 12 weeks, SVR24, SVR4, and lastly plasma HCV RNA, viral resistance and pharmacokinetics at 24 weeks. These are clearly declared as "outcomes" in the trial registry entry, and are no different in character to any other outcomes one might find on a registry.
**Multiple sets of discrepant prespecified outcomes**		
Making reference to protocols that are publicly inaccessible, or were published after trial commencement; which allegedly contain outcomes that are discrepant with registry entries but consistent with the published report.	"The trial was registered at ClinicalTrials.gov where we indicated that the primary outcome was... The protocol was also sent to The Lancet shortly after the study started and a summary of the protocol was published. The submitted protocol clearly indicated that the primary outcome was… all outcomes at 2 years prespecified in the submitted protocol were reported but were not included in the published protocol" (Trial 10, Annals, 16/04/16).	The argument appears to be that there is a publicly inaccessible pre-commencement protocol that contains prespecified outcomes different from those in the contemporaneous pre-commencement registry entry. There is no methodological justification for discrepant outcomes between registry entry and protocol for the same trial at the same timepoint: both should be the same, and changes after trial commencement should be discussed in the results paper. Registries were devised as a publicly accessible location for trial information specifically to prevent selective outcome reporting. Multiple discrepant sets of prespecified outcomes, with the option to choose between multiple discrepant documents, undermines the purpose of prespecifying outcomes.
	"We note the Correspondence by Eirion Slade and colleagues stating that we reported 21 endpoints that were not prespecified in our Article [1]. Their assertion is not correct. Our protocol, dated Oct 11, 2012, and statistical analysis plan, dated July 26, 2013, delineated secondary composite endpoints… That statistical analysis plan also specified exploratory tertiary endpoints" (Trial 30, Annals, 09/04/16).	As above. Note the protocol is referenced twice in the trialists' response but is not referenced, with no link given, and could not be found online.
	"Most of the problems that are mentioned stem from the lack of detail in pre-specified outcomes in the approved trial registry entry, rather than on a lack of transparent reporting. Full details of the pre-specified outcomes of our randomized controlled trial are provided in the trial protocol, which was published at the start of the trial in an open accessed journal." (Trial 70, BMJ, 04/02/16).	The protocol they described as "published at the start of the trial" was published in 2013, data collection began in 2012. Trialists are responsible for ensuring that information they enter for their own trial registry entries and protocols is clear.
	"Our analysis followed the original protocol agreed with the UK Medical Research Council…" (Trial 7, Lancet, 23/01/16)	The protocol they describe is not publicly available. COMPare therefore used the last pre-commencement registry entry, whose outcomes should not be discrepant with the contemporaneous protocol.
	"In our protocol, published 2 years before the main trial results,(3) we specified that the 12 months outcome on this questionnaire was our primary outcome end-point." (Trial 25, Annals, 11/12/15)	This document is from two years before the trial was published, but one year after the trial commenced. It therefore cannot, by definition, contain "prespecified" outcomes.
	"The rationale and design of the EXAMINATION trial,1 reported the primary and the secondary endpoints to be assessed at 1 year2 and every year up to 5 year follow-up. " (Trial 17, Lancet, 14/05/16)	All these protocols are either publicly inaccessible or from after the trial commencement date.

Theme	Trialists' response	Issue
	"Nevertheless, we can confirm that all recorded study outcomes were fully reported and that these are entirely consistent with the final (Research Ethics Committee approved) trial protocol and those prespecified in the study analysis plan before unblinding of data." (Trial 8, Lancet, 13/02/16)	
	"On behalf of all the authors of the NAPOLI-1 study,1 we would like to clarify that all endpoints, primary and secondary including safety, reported in our paper were prespecified and designated as such in the study protocol and statistical analysis plan." (Trial 60, Lancet, 14/05/16)	
	"There is indeed a discrepancy for the health-related quality of life assessment, which was not specified on ClinicalTrials. gov (number NCT00323960) but cited in the protocol." (Trial 71, Lancet, 25/06/16)	
Making reference to multiple discrepant sets of prespecified outcomes.	"All primary and secondary endpoints reported in The Lancet were listed at ClinicalTrials.gov before data lock and unblinding. The protocol… also posted on the public domain, EudraCT, before patient recruitment, correctly identified the primary objective… The primary outcome measure was correctly stated on EudraCT" etc (Trial 57, Lancet, 02/04/16)	This trial had multiple different sets of conflicting "prespecified" outcomes in different locations at similar dates. For example, different outcomes are registered on clinicaltrials.gov in February 2015 and July 2015; and both these sets of outcomes are in turn inconsistent with those in the protocol of June 2015.
	"stent thrombosis was prespecified and therefore reported according to its ARC categorisation as definite or probable, and acute, subacute, late, and very late." (Trial 17, Lancet, 14/05/16)	Outcomes in the trial report relating to stent thrombosis were discrepant with their original prespecified outcomes; but they were also discrepant with this section of the trialists' reply to COMPare's correction, making three discrepant sets of outcomes in total. (Outcomes reported were: stent thrombosis, definite or probable stent thrombosis, combined endpoint of all-cause death or definite stent thrombosis, combined endpoint of all-cause death or definite or probable stent thrombosis).
**Issues with timepoints**		
Incorrect statements around issue of multiple timepoints.	"We do not see how these multiple measurement time points should be counted as separate outcomes, as the procedure of the COMPare team seems to propose. We think this leads to misuse of overall statistics on their website and exaggerated conclusions about the magnitude of outcome switching in RCTs." (Trial 70, BMJ, 04/02/16)	The trial report states "Secondary outcome measure were symptoms of depression and anxiety measured with the CES-D and HADS-A at baseline and at 3, 6, 9, 12, 18 and 24 months"; and all these time-points are then separately reported in table 4 as a mean with a standard deviation. These are all outcomes, according to the CONSORT guidelines. None of these timepoints was pre-specified before trial commencement; therefore 21 non-prespecified secondary outcomes were reported.
	"...we perhaps naively assumed that we correctly completed our Registry entry by entering not just the primary measure but also the time points at which we measured it. The Registry entry does not ask for a primary end-point." (Trial 25, Annals, 11/12/15)	The prespecified outcomes were measured at multiple timepoints, none of which was given any special status. The trial report however stated that one timepoint was the "primary" outcome. Because of variation over time, and the risk of selective reporting, each separate timepoint at which an outcome is measured is an outcome, and a subset of these should be prespecified as primary, if they are reported as such.

References throughout are to the correspondence archive at COMPare-trials.org/data containing the full public correspondence on all trials, and all correspondence with editors, organised by Trial ID and date, or Journal Name for general correspondence.
