# Supplementary material for: COMPare: Qualitative analysis of researchers’ responses to critical correspondence on a cohort of 58 misreported trials
Source: Trials. 2019 Feb 14;20:124. doi: 10.1186/s13063-019-3172-3 (PMC6374909; doi:10.1186/s13063-019-3172-3)
Supplement: Supplementary file 2 — Full table of examples of trialists’ response styles. References are to correspondence archive at http://COMPare-trials.org/data. (PDF 93 kb) [file 13063_2019_3172_MOESM2_ESM.pdf]

Theme	Trialists' response	Issue
**Diversion**		
Stating that trials are hard work to conduct.	"Our 13 authors and 44 collaborators dedicated almost a decade to bringing to fruition the first prospective comparison of drug treatments for resistant hypertension." "The obstacles to performing all clinical trials these days are immense" (Trial 57, Lancet, 02/04/16)	
Stating that other issues are more important	"We also believe that larger issues are at stake in keeping control over the procedure of a pragmatic trial that merit more discussion on its influence than outcome counting, e.g. the development and implementation of interventions, training professionals to comply with strict protocols, setting up a trial in multiple centres using the same procedures, keeping contact with participants to avoid drop-out (often impossible to avoid due to illness or death), blinding of outcome assessors, medical ethics, phishing incidents [2], etcetera." (Trial 70, BMJ, 04/01/16)	
Response based on issues not raised by COMPare	"The only deviation we can see from the ISCRTN entry is the fact that we exceeded our initial trial sample size (691 in the published report versus 600 in the trial registry). We don't think this is a hanging offence, and we did this to ensure we maintained our level of pre-specified statistical power when follow up was a little lower than we anticipated (such things do happen). We note that trials commonly fail to achieve their pre-specified sample size..." (Trial 47, BMJ, 21/12/15)	All examples given here discuss issues that COMPare did not raise. None of them justify undeclared discrepancies between prespecified and reported outcomes. For trial 47, for example, the only publicly accessible pre-commencement outcomes were in the ISCRTN registry entry. This contains 11 prespecified secondary outcomes, 3 of which are not reported in the BMJ paper, with no declaration of their omission.
Ad hominem	"In the last few months, the COMPare team has monitored five top journals to analyse trials on outcome switching. Based on their interpretation of the CONSORT guidelines, comments on outcome switching have been produced. However, until now, their work has not gained or secured widespread support - neither by funders (their project is paid out-of-pocket) nor by the editors of the five top journals who do not seem keen to publish their comments... " (Trial 70, BMJ, 01/04/16) "With their approach of criticising and not being open to discussion... COMPare places themselves outside the research community. Although it can be debated to what extent it is possible to develop and criticise an aspect of science from the outside by persons not directly involved [4], we believe the research community should be critical, but with the aim to support and improve science." (Trial 70, BMJ, 01/04/16)	
**Challenging legitimacy of discussion**		
Expressing a preference for conventional peer review over open post-publication critical appraisal.	"In retrospect, we believe that expert and constructive peer reviews are sufficient to raise science to a higher level." (Trial 70, BMJ, 04/01/16)	
Disagreement with the general approach of COMPare	"The COMPare team might well catch some true outcome switching and 'fishing' ; however, in their net they are also catching researchers who have not switched outcomes or selectively reported, but have simply made minor errors of omission in their registry entries." (Trial 10, Lancet, 23/07/16)	

Theme	Trialists’ response	Issue
Asserting that there should be the opportunity to post comments on COMPare's own raw data sheets online.	"We hope the COMPare project team will take into account our comments, post our response on their website..." (Trial 17, Lancet, 14/05/16)	We set out to correct the record of misreported trials in the journal where they were misreported. Although we shared our raw underlying data sheets in an online repository we felt that the appropriate place for a critical discussion about the correct reporting of the prespecified outcomes was the journal where the trial results were reported. Consigning the discussion to our online data repository, rather than journal correspondence, would significantly reduce the visibility of a constructive discussion around correct outcome reporting.
Stating that they applaud the overall goal, followed by a caveat.	"While we support the principles of COMPare..." (Trial 25, Annals, 11/12/15)	
**"Trust the trialist"**		
Statement that discrepancies were not motivated by desire to manipulate findings.	"In response to Dale and colleagues, it should be noted that the PATHWAY programme was devised by eight academic investigators with no vested interests other than a wish to answer previously intractable questions arising from centuries of cumulative experience of hypertension practice and trials." (Trial 57, Lancet, 02/04/16)	It is unlikely that all outcome misreporting reflects a deliberate attempt by trialists’ to misrepresent a study’s findings; however a culture of permissiveness around correct outcome reporting does permit misrepresentation.
Stating that outcome misreporting doesn’t matter if the main results of the study are unlikely to be affected.	“If Dale and colleagues’ inference is that spironolactone’s overwhelming superiority over licensed antihypertensive drugs is due to selection of multiple results..” (Trial 57, Lancet, 02/04/16)	It is unlikely that all outcome misreporting changes or exaggerates the overall finding from a trial. However the evidence from the current systematic review shows that this tends to be the case, and a culture of permissiveness around correct outcome reporting facilitates such misrepresentation.
**Incorrect statements about outcome reporting in their own paper.**		
Denying that specific misreported outcomes were indeed misreported.	“We have clarified in the Methods section that physician diagnosed pneumonia was not a primary outcome”. (Trial 27, Lancet, 30/01/16)	COMPare searched the paper repeatedly and found no such disclosure; in fact the paper in question explicitly describes physician diagnosed pneumonia as the “co-primary outcome”.
General denial of COMPare's findings.	"We whole heartedly agree with the scrutiny of endpoints in high-profile clinical trials such as ours that Dale and colleagues have performed. It is reassuring that this analysis indicates that our Article is correctly reported and as such is consistent with the scientific and clinical intent of the trial as described in the protocol." (Trial 56, Lancet, 11/06/16)	This trial was not correctly reported, as explained in the COMPare letter to which this comment was a reply: two prespecified outcomes were unreported, and four additional outcomes were reported without disclose that they were novel.
**Technical / Rhetorical**		
Appealing to the existence of a novel category of outcomes whose results need not be correctly reported	"none of these are key secondary endpoints" (Trial 56, Lancet, 11/06/16)	The outcomes prespecified in the registry entry were not reported for this trial. The phrase "key secondary outcomes" is one used by WHO, in their list of 20 items that should be in all registry entries, to denote all the secondary outcomes prespecified in the registry, which should all be reported.

Theme	Trialists' response	Issue
Stating that space constraints prevent all prespecified outcomes being reported.	"As indicated by Aaron Dale and colleagues, two of three prespecified primary outcomes were not fully described in the results section of our Article for word limitation reasons." (Trial 29, Lancet, 11/06/16)	While the authors appeal to length limits, this paper reported an additional outcome ("distribution of clinical stages of cancer"), stratified by clinical stage, percentage of reported breast cancer positive patients and relative sensitivity. This resulted in their reporting 16 additional outcomes that were not pre-specified (none of which were declared as non-prespecified). Reporting non-prespecified outcomes was common throughout the project.
Stating that it is not necessary to prespecify some outcomes as they are "necessarily implied" by other outcomes.	"the adjudication of the prespecified endpoints of any myocardial infarction, target vessel myocardial infarction, revascularisation, or target vessel revascularisation, necessarily implies the assessment of the non-target vessel myocardial infarction and the non-target vessel revascularisation." (Trial 17, Lancet, 14/05/16)	This is an additional outcome that was no prespecified. Clear prespecification is required by registers, regulators and CONSORT, in order to avoid selective reporting. Unnecessary flexibility leaves trialists the option to selectively report outcomes, with no public record of the original intentions of the trial.
Inaccurate statements about COMPare's methods	"... we suggest that a trial's published protocol should also be reviewed by COMPare in tandem with its Registry entry as part of their process." (Trial 25, Annals, 11/12/15)	The COMPare method used both: preferentially protocols; and where these were unavailable, or published after trial commencement, then the trial registry entries were used instead.

References throughout are to the correspondence archive at COMPare-trials.org/data containing the full public correspondence on all trials, and all correspondence with editors, organised by Trial ID and date, or Journal Name for general correspondence.
