# Supplementary material for: COMPare: Qualitative analysis of researchers’ responses to critical correspondence on a cohort of 58 misreported trials
Source: Trials. 2019 Feb 14;20:124. doi: 10.1186/s13063-019-3172-3 (PMC6374909; doi:10.1186/s13063-019-3172-3)
Supplement: Supplementary file 3 — Full table of examples of trialists’ error acknowledgements. References are to correspondence archive at http://COMPare-trials.org/data. (PDF 84 kb) [file 13063_2019_3172_MOESM3_ESM.pdf]

Theme	Trialists' response	Issue
**Acknowledgement of error by trialists**		
Clear acknowledgement of CONSORT breach, and clarification.	"One prespecified secondary outcome from the protocol (assisted vaginal delivery) was omitted from the analysis plan in error, and, therefore, not reported" (and further corrections for same trial). (Trial 46, Lancet, 14/04/16)	We regard a clear correction as best practice. In our cohort of 58 submitted letters it was uncommon.
	"The trial was registered by the study sponsor, but we did not ensure that that registration was complete. We and our academic research groups recognise the importance of full public reporting of trial design and results, and we apologise for this oversight." (Trial 30, Lancet, 09/04/16)	Note these researchers also argued that their outcomes were correctly prespecified in a protocol from before trial commencement, and refer to this document twice in their letter; but they give no citation, and no link, and COMPare researchers were unable to find this protocol online, despit extensive searching
	The authors explained that they submitted a pre-trial protocol to the journal, which was not published, and which was discrepant with the registry entry. "When the trial was registered, only the primary endpoint (overall survival), and secondary endpoints of progression-free survival, and objective response rate were included in the ClinicalTrials.gov registry, because we did not have a complete understanding of the requirements of listing all secondary endpoints. We thank the COMPare team for bringing this inadvertent omission to our attention, and we plan to update the ClinicalTrials.gov registry as soon as possible to include all secondary endpoints prespecified in the protocol and included in our paper." (Trial 60, Lancet, 14/04/16)	The contents of this trialists' response cannot be validated as the pre-commencement protocol is not publicly accessible; furthermore, silently updating the registry entry in retrospect, so that they agree with the published report, is not relevant. However this is a clear acknowledgement and clarification on the reporting discrepancy.
	"As indicated by Aaron Dale and colleagues, two of three prespecified primary outcomes were not fully described in the results section of our Article for word limitation reasons." (Trial 29, Lancet, 11/06/16)	
	"We did not report the results of the Steatotest as we had incomplete data for this because of sample haemolysis… For the purpose of transparency, we include the median values at baseline…" "We presented data on three parameters that had not been predefined as secondary endpoints…" (Trial 56, Lancet, 11/06/16)	Note trialists also introduced a spurious distinction regarding unreported outcomes ("none of these are key secondary endpoints").
	We accept that our reporting of the change in the primary depression outcome in the BMJ paper could have been better ... we accept that, by rule, we have failed to be entirely transparent and we meet their criteria for such a rating." (Trial 47, BMJ, 14/01/16)	
	"The COMPare team is correct in that there are minor differences in the secondary outcomes in our submitted protocol and those in the trial registry." (Trial 10, Lancet, 23/07/16)	
	"Distribution of clinical stages of breast cancer is an important indicator of the primary endpoint, which is not stated in the clinical trial registry or flagged as such, as indicated by Dale and colleagues. We believe that the distribution of clinical stages affects the detection rate of breast cancer and therefore helps the reader to interpret the primary results. These results should be considered exploratory..." (Trial 29, Lancet, 11/06/16)	
	"Six primary outcomes were listed in the ISRCTN registry… and impaired activity (not reported)" (Trial 7, Lancet, 23/01/16)	CONSORT 6b states trialists should report "any changes t trial outcomes after the trial commenced, with reasons"; Trial 7 authors acknowledge non-reporting but do not give reasons [note multiple other errors and misapprehensions from Trial 7 throughout their letter].

Theme	Trialists' response	Issue
Correcting the wrong error.	"We have reviewed these discrepancies and concede that we failed to fully update the trial registry." (Trial 8, Lancet, 13/02/16)	The error was not failure to update the registry entry, but rather failure to report prespecified outcomes, or document discrepancies.
	"We acknowledge the fact that we did not update the trial information on ClinicalTrials.gov; nevertheless, all the endpoints reported were truly prespecified in the design paper." (Trial 17, Lancet, 14/05/16)	The error was not failure to update the registry entry, but rather failure to report prespecified outcomes, or document discrepancies. The design paper is from after trial commencement.
	"We probably should have mentioned the multiple measurement time points in the trial register; however, a full description can be found in our design paper giving the rationale why we used multiple measurement time points and how we modeled them longitudinally." (Trial 70, BMJ, 04/02/16)	This design paper is from after trial commencement. Changes from pre-commencement outcomes were not reported.

References throughout are to the correspondence archive at COMPare-trials.org/data containing the full public correspondence on all trials, and all correspondence with editors, organised by Trial ID and date, or Journal Name for general correspondence.
